# Impact of influenza on hospitalization rates in children with a range of chronic lung diseases

**DOI:** 10.1111/irv.12633

**Published:** 2019-01-30

**Authors:** Nusrat Homaira, Nancy Briggs, Ju‐Lee Oei, Lisa Hilder, Barbara Bajuk, Tom Snelling, Georgina M. Chambers, Adam Jaffe

**Affiliations:** ^1^ Faculty of Medicine Discipline of Pediatrics School of Women's and Children's Health UNSW Sydney Sydney New South Wales Australia; ^2^ Respiratory Department Sydney Children's Hospital Sydney New South Wales Australia; ^3^ Stats Central, Mark Wainwright Analytical Centre UNSW Sydney Sydney New South Wales Australia; ^4^ Department of Newborn Care Royal Hospital for Women Sydney New South Wales Australia; ^5^ Centre for Big Data Research in Health UNSW Sydney Sydney New South Wales Australia; ^6^ NSW Pregnancy and Newborn Services Network Sydney Children's Hospitals Network Sydney New South Wales Australia; ^7^ Princess Margaret Hospital Perth Western Australia Australia; ^8^ Wesfarmers Centre of Vaccines & Infectious Diseases Telethon Kids Institute University of Western Australia Perth Western Australia Australia; ^9^ Menzies School of Health Research Charles Darwin University Darwin Northern Territory Australia; ^10^ School of Public Health Curtin University Bentley Western Australia Australia; ^11^ National Perinatal Epidemiology and Statistics Unit (NPESU) Kensington New South Wales Australia

**Keywords:** chronic lung diseases, influenza burden, pediatrics

## Abstract

**Background:**

Data on burden of severe influenza in children with a range of chronic lung diseases (CLDs) remain limited.

**Method:**

We performed a cohort study to estimate burden of influenza‐associated hospitalization in children with CLDs using population‐based linked data. The cohort comprised all children in New South Wales, Australia, born between 2001 and 2010 and was divided into five groups, children with: (a) severe asthma; (b) bronchopulmonary dysplasia (BPD); (c) cystic fibrosis (CF); (d) other congenital/chronic lung conditions; and (e) children without CLDs. Incidence rates and rate ratios for influenza‐associated hospitalization were calculated for 2001‐2011. Average cost/episode of hospitalization was estimated using public hospital cost weights.

**Results:**

Our cohort comprised 888 157 children; 11 058 (1.2%) had one of the CLDs. The adjusted incidence/1000 child‐years of influenza‐associated hospitalization in children with CLDs was 3.9 (95% CI: 2.6‐5.2) and 0.7 (95% CI: 0.5‐0.9) for children without. The rate ratio was 5.4 in children with CLDs compared to children without. The adjusted incidence/1000 child‐years (95% CI) in children with severe asthma was 1.1 (0.6‐1.6), with BPD was 6.0 (3.7‐8.3), with CF was 7.4 (2.6‐12.1), and with other congenital/chronic lung conditions was 6.9 (4.9‐8.9). The cost/episode (95% CI) of influenza‐associated hospitalization was AUD 19 704 (95% CI: 11 715‐27 693) for children with CLDs compared to 4557 (95% CI: 4129‐4984) for children without.

**Discussion:**

This large population‐based study suggests a significant healthcare burden associated with influenza in children with a range of CLDs.

## 1 INTRODUCTION

Globally, influenza is a major public health problem. Every year during seasonal epidemics, 3‐5 million people develop severe influenza requiring hospitalization.^1^ Young children, especially those aged <5 years, are particularly vulnerable to infection; around 10%‐30% of children are infected with the virus during each influenza season.^2^ Children also play an important role in transmission by shedding large quantities of virus for 7‐10 days and introducing the virus into households.^3^

Influenza virus infection may be especially problematic in children with chronic lung diseases (CLDs). It can exacerbate the respiratory symptoms of the underlying lung condition resulting in unscheduled medical presentations and contributing to the total burden of CLDs on the health system. Children with CLDs such as asthma have been reported to be at double the risk of hospitalization with influenza compared to children with other chronic conditions.^4^ Data on the burden of influenza among children with other CLDs are more limited, and largely limited to the burden in children with cystic fibrosis (CF).^5^ Such data are crucial for informing policy makers, clinicians, and public health professionals of the magnitude of the problem and for monitoring burden of disease over time and evaluate vaccine effectiveness thus influencing influenza vaccine policy. We therefore conducted a retrospective population‐based cohort study designed to measure the incidence rates and direct healthcare cost related to influenza‐associated hospitalization in children with CLDs.

## 2 PATIENTS AND METHODS

### 2.1 Study population

The study was conducted in New South Wales (NSW), Australia, comprising all children born and residing in NSW during 2001‐2010 with follow‐up until the end of 2011 (31 December 2011).

### 2.2 Study design

The retrospective cohort study used linked population‐based administrative data sets. In NSW, The Centre for Health Record Linkage (CHeReL) (www.cherel.org.au) conducts linkage of administrative health data sets for research purposes and provides each child with a unique Patient Project Number (PPN). The de‐identified data sets with the unique PPNs were provided to the study investigators. Records of the same child in the different data sets were combined using the unique identifier key.^6^

The NSW Perinatal Data Collection (PDC) which records all births in NSW was used as the primary data source for identifying the study cohort. Information relating to maternal and child factors such as maternal smoking during pregnancy, number of previous pregnancies, Indigenous status of the mother, postcode of area of residence, birth weight, gestational age at birth, and sex of the baby was ascertained from PDC. Data relating to diagnosis of CLDs and influenza‐associated hospitalization were ascertained from the Admitted Patient Data Collection (APDC) and Neonatal Intensive Care Unit Data (NICU). The data sets have been described previously.^6^

### 2.3 Exposure assessment

The birth cohort was divided into five mutually exclusive sub‐groups. We used the International Classification of Diseases. 10th edition (ICD‐10), primary diagnostic codes to identify children in each sub‐group, which was introduced in Australian hospitals in July 1998.^7^

1. Children with severe asthma: Children aged >2 years who had ≥2 asthma hospitalization after the age of 2 years with ICD‐10 diagnostic codes associated with asthma (J45), predominantly allergic asthma (J45.0), non‐allergic asthma (J45.1), mixed asthma (J45.8), asthma unspecified (J45.9), and status asthmaticus (J46). As diagnosis of asthma is difficult in children aged <2 years 8 and as the national immunization guidelines recommend seasonal influenza vaccine for children with severe asthma (requiring frequent hospitalization),^9^ only these children were included in the analysis. Additionally, we excluded ICD codes associated with recurrent wheeze as many of these could have been due to respiratory viral infection and not due to asthma.
2. Children with bronchopulmonary dysplasia (BPD): Children who were born at gestational age (GA) ≤32 weeks and required oxygen and/or any type of respiratory support at 36 weeks’ GA 10 or had any history of hospitalization with ICD‐10 diagnostic code associated with bronchopulmonary dysplasia originating in the perinatal period (P27.1). The NICU data set and the APDC data sets were used to identify children with BPD.
3. Children with CF: Children with any history of hospitalization with ICD‐10 diagnostic codes associated with CF (E84), CF with pulmonary manifestations (E84.0), CF with intestinal manifestations (E84.1), CF with other manifestations (E84.8), CF unspecified (E84.9).
4. Children with other congenital and chronic lung conditions: Children with any hospitalization code associated with congenital/chronic lung conditions for which there was an available ICD code including congenital diaphragmatic hernia (Q79.0‐Q79.4), congenital tracheo‐esophageal fistula (Q39.2‐Q39.8), congenital tracheomalacia (Q32.0‐32.4), congenital malformation of lung and respiratory system (Q33‐Q34), bronchiectasis (Q33.4), dependence on ventilators (Z99.1), interstitial emphysema (J98.2), other specified and unspecified interstitial lung diseases (J84.8 and J84.9), and Kartegener's syndrome (Q89.3).
5. All other children without chronic lung diseases.

### 2.4 Primary outcome

Influenza‐associated hospitalization: The ICD‐10, primary diagnostic codes associated with influenza‐associated hospitalizations were used to identify all influenza‐associated hospitalizations from APDC. Any ICD‐10 diagnostic codes listed as influenza and pneumonia (J09, J10, J10.0, J10.1, and J10.8) where influenza virus was identified were considered to be associated with influenza. All other hospitalizations associated with influenza and pneumonia code but where a virus was not identified (J11.0, J11.1, and J11.8) and where the hospitalization occurred during influenza season (usually between May‐September in Australia) were also considered to be associated with influenza.

### 2.5 Other co‐variates

Data on maternal and child factors considered to be independent risk factors for acute lower respiratory infections 11 including multiparity of the mother (previous pregnancy lasting >20 weeks), maternal smoking during pregnancy, Indigenous status of the mother, residential postcode of the mother at birth, small for gestational age, and sex of the cohort child were ascertained from the PDC. Frequency of all‐cause‐associated hospitalizations in the cohort child was retrieved from the APDC. Socioeconomic disadvantage was inferred from the maternal postcode using the Socioeconomic Index of Areas (SEIFA) and Index of Relative Socioeconomic Advantage and Disadvantage (IRSAD) compiled by the Australian Bureau of Statistics.^12^

### 2.6 Bias

This was a large population‐based cohort study with minimum selection bias.

### 2.7 Study size

This was a whole‐of‐population study including all children born in NSW between 2001 and 2010.

### 2.8 Analyses

The overall and year‐specific incidence rates for influenza‐associated hospitalizations were calculated for years 2001‐2011. We calculated incidence as the number of new influenza‐associated hospitalizations divided by the child‐years at risk in each of the disease groups. A lag of 14 days between two successive influenza‐associated hospitalizations was considered to be a new hospitalization. We used Poisson estimation to calculate incidences and incidence rate ratios (including 95% confidence interval around the estimates) of influenza‐associated hospitalizations. The incidence rates and rate ratios were adjusted for parity of the mother, maternal smoking during pregnancy, Indigenous status, IRSAD, sex of the child, small for gestational age, and frequency of previous hospitalizations in the cohort child. There were 0.5% missing data for variables including Indigenous status of the mother, socioeconomic disadvantage of the area of residence, and maternal smoking during pregnancy. Observations with one or more variables missing were dropped from the analyses.

Inpatient hospital costs associated with influenza was estimated using NSW Cost of Care Standards 2009/10.^13^ These standards specify costs and cost weights based on Australian Refined Diagnosis Related Group Version (AR‐DRG) for each episode of acute admitted hospital services. The cost weights provide a measure of resource consumption relative to a reference value of one in NSW representing the average inpatient hospital (public and private) admission in a given year. At discharge, each episode of acute hospital care is assigned an AR‐DRGs and associated cost weight which represents its relative resource consumption relative to the reference value. The cost weights account for admission through emergency department presentation and admission in to the intensive care unit. Further adjustments are made to the cost weights to account for same‐day admissions, extended length of stay, transfer episodes, Indigenous status, private hospital stays, neonates, and death.

To estimate hospital costs for influenza admissions, we assigned the weighted “total standard NSW public hospital cost” to the cost weights assigned to each episode of acute hospital care in the APDC using the NSW Costs of Care Standards 2009/10.^13^ The average costs for acute admitted care were 4280 Australian dollars (AUD) for 2009/10 which was indexed for each study year (average annual discount rate of 3.5%) using the deflators specified in the NSW Costs of Care Standards to reflect constant 2009/10 Australian dollars. For hospitalizations that resulted in transfer to other facilities, the cost was equivalent to the sum of total cost incurred at each of facility. We divided the total cost of all influenza‐associated hospitalization identified over the 11‐year period by 11 to estimate the annual direct cost of influenza‐associated hospitalizations. All analyses were done using STATA (STATA release 13; StataCorp LP, College Station, TX, USA).

### 2.9 Ethics approval

The project was approved by the NSW Population and Health Service Research (HREC/09/CIPHS/33; 2009/05/155) and the Aboriginal Health and Medical Research Council Ethics (726/10).

## 3 RESULTS

### 3.1 Profile of the Cohort

The cohort comprised 888 157 children born between 2001 and 2010. Of these, 11 058 (1.2%) had one of the CLDs, and 6724 (61.0%) were male. Around 4.0% of all children with CLDs were of Indigenous origin (Table 1).

**Table 1:** Descriptive profile of the cohort children born between 2001 and 2010 in NSW, Australia

N = 888 157					
Exposures	Children with severe asthma	Children with BPD	Children with CF	Children with other congenital and chronic lung conditions	All other children without chronic lung diseases
	n = 7736	n = 1055	n = 260	n = 2007	n = 877 099
	n (%)				
Multiparity of the mother	4446 (57.5)	510 (48.3)	137 (52.7)	1161 (58.0)	510 288 (58.2)
Maternal smoking during pregnancy	1315 (17.0)	253 (24.0)	41 (15.8)	330 (16.4)	120 397 (13.7)
Indigenous status of the mother	291 (3.8)	68 (6.5)	13 (5.1)	91 (4.6)	29 228 (3.4)
IRSAD					
1 (most disadvantaged)	1990 (25.7)	268 (25.5)	56 (21.5)	469 (23.4)	195 668 (22.3)
2	1893 (24.5)	229 (22.0)	64 (24.6)	486 (24.2)	202 308 (23.1)
3	1979 (25.6)	263 (25.0)	74 (28.5)	495 (24.7)	223 744 (25.5)
4 (most advantaged)	1873 (24.2)	290 (27.6)	66 (25.4)	553 (27.6)	254 923 (29.1)
Male sex of the baby	4839 (62.5)	595 (56.4)	126 (48.5)	1164 (58.0)	449 943 (51.3)
Small for gestational age at birth	320 (4.1)	56 (5.3)	14 (5.4)	147 (7.4)	28 080 (3.2)

BPD, Bronchopulmonary dysplasia; CF, Cystic fibrosis; IRSAD, Index of Relative Socioeconomic Advantage and Disadvantage.

### 3.2 Incidence of influenza‐associated hospitalization

**Figure 1:**
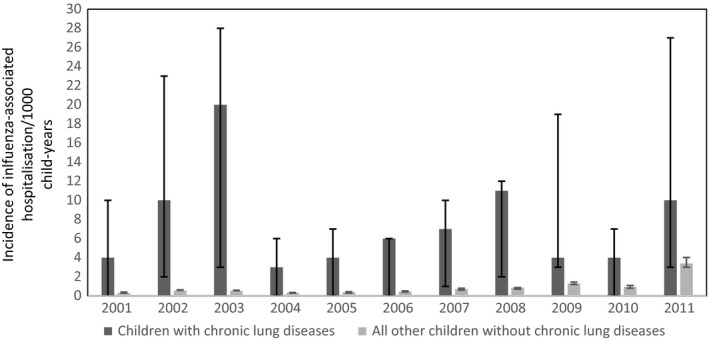
Annual incidence/1000 child‐years of influenza‐associated hospitalization in children with and without chronic lung diseases, 2001‐2011, NSW, Australia

During 2001‐2011, there were 3.101 influenza‐associated hospitalizations, and 2.4% of the children with CLDs compared to 0.3% children without CLDs had one or more influenza‐associated hospitalizations. The unadjusted and adjusted incidence/1000 child‐years of influenza‐associated hospitalization in children with CLDs was 3.7 (95% CI: 3.0‐4.3) and 3.9 (95% CI: 2.6‐5.2) and for all other children without CLDs was 0.5 (95% CI: 0.4‐0.5) and 0.7 (95% CI: 0.5‐0.9), respectively. During 2001‐2011, the annual incidence of influenza‐associated hospitalization in children with CLDs ranged between 0.0 and 25.4/1000 child‐years (Figure 1). The adjusted incidence rate ratio for influenza‐associated hospitalization in children with CLDs compared to children without CLDs was 5.4.

The unadjusted incidence/1000 child‐years of influenza‐associated hospitalization in each CLD group was 1.1 (0.7‐1.4) for children with severe asthma, 5.4 (3.6‐7.2) for BPD, 6.9 (2.5‐11.4) for CF, and 7.7 (5.9‐9.4) for children with other congenital and chronic lung conditions. The adjusted age‐specific incidence rates and rate ratios are presented in Table 2.

**Table 2:** Adjusted incidence/1000 child‐years and incidence rate ratios of influenza‐associated hospitalization in children with chronic lung diseases, 2001‐2011, NSW, Australia

Incidence/1000 child‐years (95% CI)					Incidence rate ratio (95% CI)^a^
	0‐24 mo	2‐5 y	5‐10 y	Overall incidence	
Severe asthma	N/A	5.4 (1.4‐9.4)	1.2 (0.3‐2.0)	1.1 (0.6‐1.6)	1.8 (1.2‐2.6)
BPD	41.6 (15.7‐67.5)	1.7 (−0.8 to 4.2)	1.0 (−0.8 to 4.4)	6.0 (3.7‐8.3)	9.0 (6.4‐12.7)
CF	44.5 (6.0‐83.0)	4.6 (−2.15 to 11.4)	0 (0‐0)	7.4 (2.6‐12.1)	11.12 (6.0‐20.9)
Other congenital and chronic lung conditions	42.9 (18.1‐67.8)	6.0 (1.4‐10.6)	1.1 (0.2‐2.1)	6.9 (4.9‐8.9)	10.4 (7.9‐13.5)
All other children without chronic lung diseases	9.3 (4.4‐14.2)	0.6 (0.3‐1.0)	0.1 (0.0‐0.1)	0.6 (0.4‐0.9)	Reference group

BPD, Bronchopulmonary dysplasia; CF, Cystic fibrosis; IRSAD, Index of Relative Socioeconomic Advantage and Disadvantage; 95% CI, 95% Confidence interval.

a The rate of influenza‐associated hospitalization in each of the groups of children with chronic lung diseases was significantly higher (*P* < 0.05) compared to rate in all other children without chronic lung diseases.

### 3.3 Inpatient resource consumption of influenza illness

**Figure 2:**
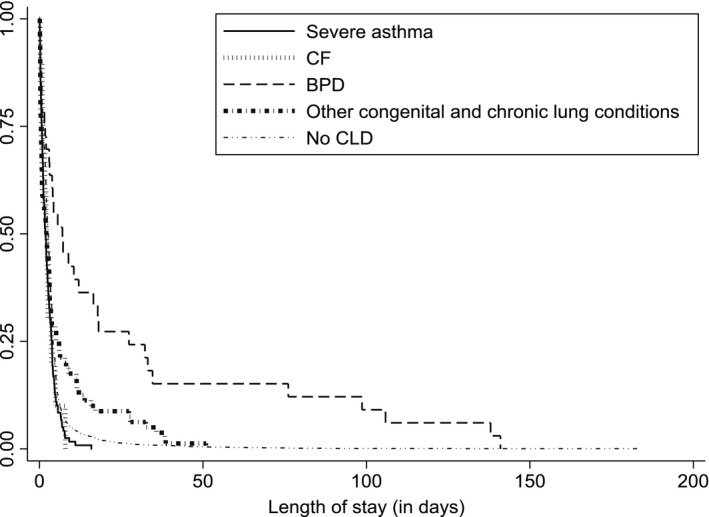
Length of stay for influenza‐associated hospitalization in the cohort children, 2001‐2011, NSW, Australia

The length of stay (days) for each episode of influenza‐associated hospitalization in children with and without CLDs is presented in Figure 2. The longest median length of stay was for children with BPD (7.0 days; IQR 1.7‐27.3). A total of 13% of the episodes of influenza‐associated hospitalization in children with CLDs required referral to another hospital for continuity of care compared to 7% in children without CLDs. The average cost/episode of influenza‐associated hospitalization for children with CLDs was AUD 19 704 (95% CI: 11 715‐27 693) equating to an average annual cost to the NSW hospital system of AUD 428 132 (equivalent to approximately US$ 314 902) and for children without CLDs was 4557 (95% CI: 4129‐4984) which was equivalent to AUD 867 033 annually (approximately US$ 637 808).

## 4 DISCUSSION

This large population‐based study has demonstrated that children with CLDs are at least five times more likely than children without CLDs to be hospitalized with influenza. A previous study from the USA has also demonstrated that children with acute cardio‐pulmonary diseases were 2‐4 times more likely to be hospitalized with influenza‐associated illness than other children.^4^ The direct medical cost of AUD 19 704/episode of influenza‐associated hospitalization in children with CLDs was also four times higher compared to children without, representing a high economic burden on the healthcare system. Furthermore, the hospitalization rates in our cohort were highest in the youngest children and decreased with age, a finding that is consistent with the USA study.^4^ Younger children especially those aged <2 years are at higher risk than older children of severe influenza,^14^ which is increased in the presence of an underlying CLD. Vaccination against influenza is the primary strategy to control seasonal outbreaks. Influenza vaccination has been proven to be a safe and effective in people with CLDs.^15^ The Australian immunization guidelines emphasize the need for seasonal influenza vaccine for people aged ≥6 months with chronic medical conditions.^9^ Maternal immunization to protect infants in their first 6 months of life 16 followed by active immunization annually may help lower the exceptionally high burden on the health system associated with influenza illness in young children with CLDs.

The rate of influenza‐associated hospitalization in children aged 2‐5 years with asthma in our cohort was much higher than the reported annual rate of 0.6/1000 person‐years in children with asthma in the USA.^17^ This could be due to differences between the study populations; the study from USA only included children who were hospitalized with acute respiratory infection or fever whereas we considered any hospitalization with ICD‐10 codes associated with influenza. Additionally, Australia has a universal healthcare system where most inpatient care for children is covered by federal state governments. Australia's immunization guidelines emphasize the need for seasonal influenza vaccination for children with severe asthma requiring frequent hospitalizations.^9^ Although the national vaccine uptake data for asthmatic children are limited, a survey conducted by Asthma Australia in 2016 demonstrated that 41% of people with asthma (mostly children) were not likely to be vaccinated against influenza clearly indicating very low uptake which is unacceptable for any other pediatric vaccine.

Children with CF in our cohort were at least 11 times more likely to be hospitalized with influenza‐associated illness compared to children without CLDs. While much attention is given to bacterial infections in children with CF, respiratory viral infections have also been associated with pulmonary deterioration and disease progression.^18^ Studies from the USA have associated increases in influenza transmission with increased frequency of pulmonary exacerbations 19 and found comparable high rates of influenza‐associated hospitalization (12.97/1000 person‐year) among children aged 0‐17 years with CF.^5^ Influenza vaccine is widely recommended for people with CF, and the vaccine has been shown to be safe in these patients 20 and, in contrast to children with asthma, the uptake of seasonal influenza vaccine has been reported to be high (≥80%).^21^, ^22^ There is a need for further research into the impact of regular seasonal influenza vaccine on pulmonary function in patients with CF.^23^, ^24^ Additionally, there is a need to examine the benefit of timely use of antivirals in these children with proven or suspected influenza infection in reducing lung damage.^20^

In our study, children with BPD also had very high rates of hospitalizations associated with influenza. The median length of hospitalization was also very long for children with BPD. Studies suggest that although symptoms of BPD improve with age, children with BPD continue to have abnormal lung function and are at elevated risk of being hospitalized with respiratory illness compared to other children.^25^, ^26^, ^27^ While several studies have shown the significant burden of respiratory syncytial virus among children with BPD,^6^, ^28^ the impact of influenza illness remains less clearly defined.

Our study also showed that the rate of influenza‐associated hospitalization was 10 times higher in children with other congenital and chronic lung diseases than all children without CLDs, yet there are almost no available data on the burden of influenza in these children.

Our study has several important limitations. There are no national disease registries for children with CLDs (with the exception of CF) so our cohort was constructed from a comprehensive hospitalization dataset based on ICD codes. Ascertainment of influenza‐associated hospitalization was also based on ICD codes. Even though not all influenza‐associated hospitalizations are laboratory confirmed but as influenza is a notifiable condition in NSW, it is likely that most hospitalizations receiving an influenza‐specific code were laboratory confirmed; however, we were not able to substantiate this. Not all children hospitalized with acute lower respiratory illness are routinely tested for influenza so it is likely that the true burden of influenza‐associated hospitalization in children with CLDs is even higher than our estimates. We considered all influenza‐coded hospitalizations where an influenza virus was not identified to be associated with influenza which may have inflated our estimates; however, none of the influenza‐associated hospitalization in our cohort was associated with an ICD code for influenza where an influenza virus was not identified. We did not have access to data on primary care or emergency presentations and therefore we could not assess the rate of ambulatory influenza infection in children with CLDs, where the burden is likely to be higher. Although we adjusted our estimated rates for a number of known potential confounders, we did not have access to direct information on other potential confounders such as household exposure to tobacco smoke or presence of siblings at home. Instead, we used maternal smoking during pregnancy as a proxy for household smoke exposure and previous pregnancy/parity (lasting at least 20 weeks) as a proxy for having older siblings at home. We used the IRSAD score of the mother's postcode at the time of delivery, a measure of average socioeconomic disadvantage in that postcode, as a proxy for family level socioeconomic disadvantage.^29^ We estimated incidence rate ratio for influenza‐associated hospitalization in children with CLDs compared to all other children which included children with other chronic conditions and may have led to lower rate ratios. Influenza vaccination status is not routinely recorded in the national immunization data base, and we did not have access to antiviral use in these children.

In summary, although only around 1.4% of the total pediatric population in our cohort had one of the chronic lung diseases, these children form a special group because of their ongoing need for healthcare services. Our data clearly show that influenza illness in these children add to the existing burden of chronic diseases on the health system. While there are no effective vaccines or antivirals for most childhood respiratory viral infections, fortunately we do have a vaccine against influenza. There are also effective antivirals which, if administered in a timely manner, may reduce disease severity.^30^, ^31^, ^32^ However, such high burden of hospitalizations indicates that current efforts at influenza prevention are inadequate among children with CLDs. Further studies investigating the effectiveness of newer vaccines, treatment, or chemoprophylaxis in these children will help lower the burden of disease.

## CONFLICT OF INTEREST

The authors have no conflicts of interest relevant to this article to disclose.
